# Occupational Exposure and Risk Assessment of Formaldehyde in the Pathology Departments of Hospitals

**DOI:** 10.31557/APJCP.2020.21.5.1303

**Published:** 2020-05

**Authors:** Elham Yahyaei, Behzad Majlesi, Mohammad Naimi joubani, Yasaman Pourbakhshi, Samira Ghiyasi, Mehdi Jamshidi Rastani, Mahmoud Heidari

**Affiliations:** 1 *Department of Occupational Health, School of Health, Guilan University of Medical Sciences, Rasht, Iran. *; 2 *Department of Management, West Tehran Branch-Islamic Azad University, Tehran, Iran. *; 3 *Department of Environmental Health Engineering, School of Health, Guilan University of Medical Sciences, Rasht, Iran. *; 4 *Institute of Medical Advanced Technologies, Guilan University of Medical Sciences, Rasht, Iran. *; 5 *Department of Environmental Engineering, Central Tehran Branch, Islamic Azad University, Tehran, Iran.*; 6 *Department of Occupational Health, School of Health, Shahid Beheshti University of Medical Sciences, Tehran, Iran. *; 7 *Research Center of Health and Environment, Guilan University of Medical Sciences, Rasht, Iran. *

**Keywords:** Formaldehyde, risk assessment, occupational exposure, pathology departments, hospital

## Abstract

**Background and Objective:**

Exposure to formaldehyde has adverse effects on health both acutely and over the long term (e.g., carcinogenicity). The substance is widely used in pathology and histology departments. This study focused on cancer risk of formaldehyde in pathology department of five hospitals in Rasht.

**Materials and Methods:**

Sampling and determination of formaldehyde in pathology department were carried out based on the NIOSH method of 3500. The working condition and working environment were investigated and a semi quantitative risk assessment were used to health risk assessment of formaldehyde and The individual lifetime cancer probability, which is defined as the increase in the probability of developing cancer during continuous exposure to an air pollutant were used to assess health risks with formaldehyde.

**Results:**

The results showed that the exposure level of all subjects were higher than the Occupational Exposure Limit for 8 hours exposure time of formaldehyde. However, in the five occupational groups, the highest weekly exposure index was observed for the Lab Technicians (0.664 ppm) at Hospital no. 5, which could have been due to more daily working hours at this sampling site and a lack of adequate ventilation. The formaldehyde concentration was in the 0.0192 to 0.326 ppm ranges for five hospital pathology departments. The cancer risk ranged from 9.52×10^-5^ to 1.53×10^-3^, and it was greater than the WHO acceptable cancer risk level.

**Conclusions:**

The results of the risk assessments can be used for managing the chemical exposure of allocated resources for defining control actions. This proce**ss **plays an important role in reducing the level of exposure to formaldehyde in pathology departments.

## Introduction

Formaldehyde (HCHO), also known as formalin, methanal, formal, and methylene oxide, is an organic carbonyl compound that is colorless, is flammable, and has an irritatingly pungent gas/vapor odor in the aliphatic series (Shaham et al., 2002; Santovito et al., 2011). It has high solubility in water and high reactivity (Orsiere et al., 2006; Salthammer et al., 2010). Its high water solubility makes this compound easily distributed in the human body. Formaldehyde also is an important precursor to many other materials and chemical compounds. Because of these properties, exposure to formaldehyde has adverse effects (Jalili et al., 2019; Barkhordari et al., 2017; Attari et al., 2015) on health, including acute effects (e.g., bronchial asthma (Suzuki et al., 2017), chemosensory irritation (Bellisario et al., 2016; Persoons et al., 2011; Vimercati et al., 2010), and respiratory symptoms (Corradi et al., 2012; Afshari et al., 2014; Sekine and Nishimura, 2001), carcinogenic effects (e.g., nasopharyngeal cancer (Orsiere et al., 2006; Berton and Novi, 2012), limited evidence for leukemia and sinonasal cancer (Pala et al., 2008; Naya and Nakanishi, 2005; Driscoll et al., 2016), and genotoxic damage (Mirabelli et al., 2011; Speit et al., 2012; Bi et al., 2017; Tompa et al., 2006; Musak et al., 2013).

According to the International Agency for Research on Cancer (IARC), formaldehyde is classified as a human carcinogen (group 1) (Orsiere et al., 2006; Persoons et al., 2011). However, its effects depend on the concentration and length of exposure to it. According to the Occupational Safety and Health Administration (OSHA), the recommended permissible exposure limit of formaldehyde is 0.75 ppm (8-hour time-weighted average [TWL]) and the short-term exposure limit (STEL) is 2 ppm. The recommended exposure limit (REL) of the National Institute for Occupational Safety and Health (NIOSH) for formaldehyde is 0.016 ppm (8-hour TWL) (Vohra, 2011).

Formaldehyde widely used in the production of resins and construction materials, and is used for a variety of other purposes in hospitals and other industries (Zhou et al., 2011). Environmental sources of formaldehyde consist of building materials, engine exhaust, combustion processes, tobacco smoke, incinerators, and motor vehicle exhaust (Bruno et al., 2018). In the medical field, formaldehyde is widely used in pathology or histology departments and in autopsy rooms for sterilizing, and as a preservative (formalin) or dehydrating agent during mixture preparation, tissue processing, and staining (Corradi et al., 2012). Nurses and clinicians have different occupational and ergonomic problems (Afshari et al., 2014). However, formaldehyde poses a health risk for nurses, clinicians and physicians (Elshaer and Mahmoud, 2017) but even more so for those employed in the healthcare industry as these individuals may be exposed to formaldehyde, in addition to other various genotoxic substances. So, the risk assessment of occupational exposure to chemicals is an important step in chemical monitoring and control in pathology departments. 

## Material and Mthods

*Sampling Sites*

The study was conducted in five major hospitals in Rasht, Iran and their associated pathology departments were selected for study. The five occupations typically found in a pathology departments include pathologist, lab technician, office worker, housekeeping staff, and lab workers, and most have about 65 employees. The sampling was performed from August to September 2018 (8:00 and 12:00 AM). Formaldehyde was used in liquid form (formalin) at these sites. Additionally, the demographic and workplace environment information were collected (Table 1). 

*Environmental monitoring*

To measure occupational exposure concentrations of formaldehyde in the pathology departments of the five hospitals, personal sampling was carried out in the breathing zone of workers, hospital staff, and nurses, during formaldehyde related tasks (8 hours’ time-weighted average). The National Institute for Occupational Safety and Health (NIOSH) standard method (NIOSH 3500) was used for exposure monitoring of formaldehyde. For accurate determination of time-weighted average of exposure to formaldehyde in pathology departments, it is important to determine the concentration for exposure in tasks in which the operator directly worked with formaldehyde, as well as during that time when the presence of formaldehyde in a department’s atmosphere exposed the operator for an extended period of time at a low concentration. For this reason, personal sampling was performed in the periods with direct exposure to formaldehyde (25 min for each task), environmental sampling for a department’s ambient air for the whole of working shift (8 hours) as background exposure was taken, and finally a time-weighted average concentration was calculated for each operator. The samples were collected by personal sampling pump (SKC Universal PCXR8 Sample Pump Single Kit) at 0.8 Lmin-1 flow rate with PTFE (Polytetrafluoroethylene) membrane filter (37 mm) and dual impingers in series. Sampling was performed 3 times and kept in polyethylene bottles for further analysis.

*Analytical method*

Based on NIOSH 3500 analytical standard method, 8 level of concentration (0.04-0.8 mg/L) of formaldehyde was prepared for plotting the calibration curve. For this step, a calibration stock solution by dilution of 1 mL of 1 mg/mL formaldehyde stock solution to 100 mL 1% sodium bisulfite solution was prepared. Then the amounts of 0.1, 0.3, 0.5, 0.7, 1.0, 1.5, 2.0 and 3.0 mL calibration stock solution were diluted in 25-mL glass-stoppered flasks. To analyze the samples collected in the PTFE membrane filter, The UV/VIS spectrophotometer (DR5000HaCH) was used. Quantification of formaldehyde was carried out by adding chromotropic acid (0.1 mL) as reagent solution and sulfuric acid was added (6 mL) as a solvent.

*Risk assessment*

Based on a semi-quantitative chemical risk assessment provided by the Department of Occupational Health and Safety of Singapore, this cross-sectional study was pursued as a health-related risk assessment for exposure to formaldehyde in the pathology departments of five hospitals the method for risk assessment of harmful substances was performed as follows: 

1. In the first step, a workgroup was formed with three industrial hygienists and three highly experienced nurses, as well as one expert heading each of the pathology departments. The details pertaining to working processes, sampling strategies, and risk assessment methods were discussed. 

2. All tasks of the pathology department for each hospital were analyzed, i.e., time periods for task completion, number of persons collaborating, etc. For accurate estimation of exposure to formaldehyde. 

3. Determination was made of the hazard rate of formaldehyde based on the amount or toxicity risks and carcinogenicity of this compound. The Singapore method of chemical risk assessment introduces a scale for determination of hazard rate of substances based on chronic and acute effects as follows: 

A) Obtaining the hazard rate with the use of toxic or harmful effects of formaldehyde (Table 1). 

B) Determining the hazard rate to the acute toxicity of chemicals by determining the lethal dose (LD50) and lethal concentration (LC50) extracted from the MSDS chemicals (Table 2). 

4. Interviews with personnel of pathology departments about their working conditions and subsequent task analysis to determine the amount, frequency, route and duration of exposure of pathology departments to formaldehyde and using these factors to calculate the exposure rate when the exposure monitoring results are not available. 

5. Determining the exposure rate (ER) for formaldehyde in the following ways: 

A) Determining of the exposure rate using the actual level of exposure: When the results of measuring the concentration of chemical substances (air monitoring) are available, the mean weekly exposure to chemical agents using the following equation can be obtained. 

In the equation (1), it is assumed that when at rest (when a job duty is not done) the person is not dealing with chemicals. After calculating the weekly average exposure (E) according to the Table 3, the exposure rate can be determined using equation (1). 

$E=\frac{F\times D\times M}{W}$

 Eq.1

**Figure 1:**
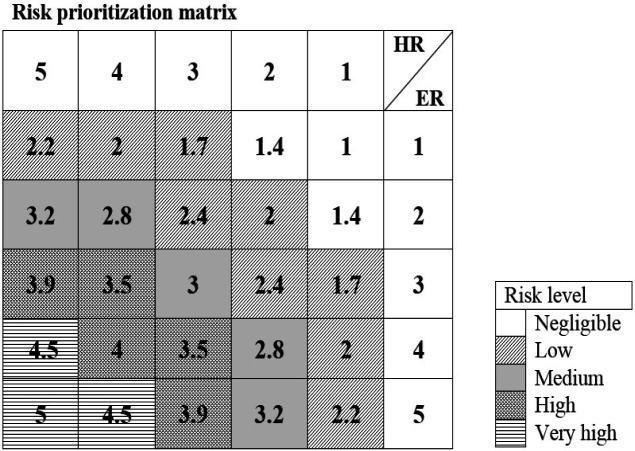
Risk Prioritization Matrix Based on Hazard and Exposure Rate of Each Chemical Compounds

Where E is the weekly exposure to each chemical compounds (ppm or mg/m3), W is average hours worked per week (40 hours), D is the average time of exposure to chemical substance, F is the exposures frequency in the time period of a week, M is concentration of exposure to the chemical substances (ppm or mg/m^3^). After determining the average exposure to chemical substance, the proportion of E to permissible exposure limit (PEL) should be determined and then the exposure index for predetermined exposed concentration to each harmful chemicals can be determined using Figure 1.

B) Determining the exposure rate when air sampling was not performed and real concentration for exposure to each chemical compounds was not determined. In this situation, the exposure index should be evaluated by estimation and accurate analysis of working condition. In the event that the results of air monitoring (measurement of exposure value) is not available, the exposure rate can be achieved through the following equation: 

Exposure index is obtained in terms of a rating 5 each (from one to five) and according to Table 4 where in 1: negligible, 2: low, 3: medium, 4: high, and 5: very high. In this study, four factors, exposure to steam pressure, control measures, the amount used in the week and working time per week were used. 

Formula (2):

$\mathrm{ER}={\left[{\mathrm{EI}}_{1}\times {\mathrm{EI}}_{2}\times \ldots {\mathrm{EI}}_{n}\right]}^{\frac{1}{n}}$

Eq.2

Where n is the number of exposure factors used, 

6. Calculating the risk factor according to the following equation: 

Formula (3): 

$\mathrm{RR}=\sqrt[{2}]{\left(\mathrm{HR}\times \mathrm{ER}\right)}$

 Eq.3

Rl: Risk rate

HR: Hazard rate (scale of 1 to 5 in Table 1 and 2) 

ER: Exposure rate (scale of 1 to 5 in Table 3).

7. Risk ratings based on risk ranking matrix in Figure 1 (Manpower 2005). To rank the risks for formaldehyde exposure the levels of negligible (N), low (L), medium (M), high (H) and very high (E) scales were used. 

In this study, LD50 and carcinogenic (ACGIH and IARC) index and the risk of corrosion was used to calculate the hazard rate and the biggest index was used as the basis of hazard rate. For calculating the exposure rate, two methods of exposure index and the actual level of exposure were used. And after determining risk by equation (3) of available risks were prioritized by risk ranking matrix (Figure 1). In the present study, the risks that were in group H and E were considered as high risk, in other words, cut of point was identified risks of group M, finally, recommendations were provided to control and reduce risks to an acceptable level.

*Estimate the individual lifetime cancer probability (LCP)*

The present study focuses on estimating the excess individual lifetime cancer probability (LCP) and identifying health hazard indices to conduct the primary health risk assessment of formaldehyde. LCP is defined as the increase in the probability of cancer occurring against a background of continuous exposure to formaldehyde. The LCPs are assessed using inhalation unit risk (IUR) estimates (µg/m^3^) for each carcinogen. The IUR estimates are defined as the individual lifetime excess risk because of a chronic lifetime exposure to one unit of pollutant concentrations (1 µg/m^3^).

*Evaluation of the cancer risk*

In the present study, the individual lifetime cancer probability (LCP) is defined as the increase in the probability of cancer during exposure to the air pollutant continuously was used to health risk assessment of formaldehyde. LCP determination by RFA and HIFA.

R_FA_= C_FA_ × IUR_FA _× L_worker _Eq.4

Where RFA is the excess LCP for formaldehyde (FA), CFA is the concentration in µg/m3 of formaldehyde (8 hours TWA), IURFA is the IUR factor for formaldehyde, and L_worker_ is the adjustment factor for the ratio of the workplace time to 70 years. Inhalation unit risk of formaldehyde is 1.3-10^-5^ [LCP/ (µg/m^3^)]. 

In this study, the L_worker _was calculated based on the employees work 8 h per day, 5.5 days per week, 45 weeks per year, and work 35 years at the same location over a 70-year time period.

$L_{\mathrm{worker}}=\frac{35_{y}\times \frac{8h}{24h}\times \frac{5.5d}{7d}\times \frac{45w}{52w}}{70_{y}}=0.113$

Eq.5

*Evaluation of the non-cancer risk*

The hazard index (HI) show non-cancer health risks, calculate based on reference concentration (RfC) and usually compared with 1. The HI of chronic non-carcinogenic effects is calculated from equation 3.

HI_FA_=CFA / Rfc_FA_

 Eq.6

Where the RfCFA is the inhalation reference exposure level for chronic non-cancer health effects of formaldehyde that is 3.6 gr/m^3^.

## Results

The five occupations in the pathology department include pathologist (n=8), laboratory technician (n=10), office worker (n=7), housekeeper (n=32) and laboratory worker (n=5), for a total of 62 employees. Table 1 shows the workplace environment information.

Standard formaldehyde was prepared in the range of 0.005–3.0 ppm. A calibration curve with proper linearity was obtained (R^2^=0.9976) to determine the formaldehyde concentration after sampling using a standard method (Figure 1A). 

The formaldehyde concentration was in the 0.0192 to 0.326 ppm ranges for five hospital pathology departments. A comparison of the formaldehyde concentration in the five different sampling sites showed that the highest concentration was observed in pathology laboratory No. 2. The results are shown in Figure 1B.

**Figure 2:**
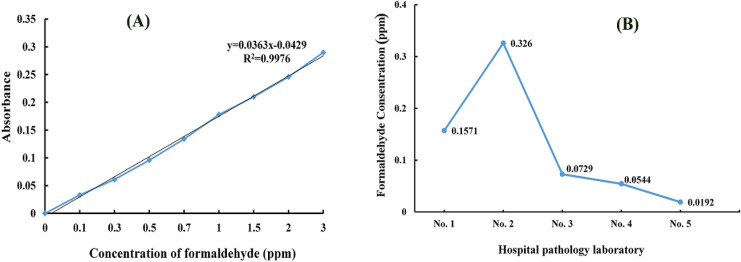
Calibration Curve of Formaldehyde Standard Concentration (A) and comparison of formaldehyde concentration measured at five different sampling sites (B)

Figure 2A shows the employee exposures in five different hospital pathology departments and different occupations based on Eq. 2. According to these results, there was a higher exposure to formaldehyde in Lab No.1 and Lab No.2. Also the pathologists and laboratory workers had a lower exposure to formaldehyde. A comparison of the employee exposures for five different occupations in five different hospital pathology departments (the weekly mean level) is also demonstrated in Figure 2B. 

Tables 2 and 3 show the results of the carcinogenicity and non-carcinogenicity risk assessment of the employee exposures according to the type of occupation from each of the sampling sites separately.

**Figure 3:**
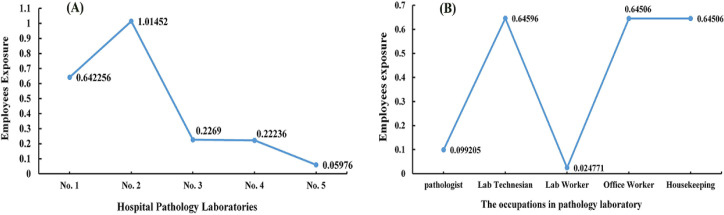
Comparison of Employees Exposure at Five Different Sampling Sites (Weekly mean Level) (A) and Comparison of Employees Exposure at five different Occupations (Weekly mean Level) (B).

Figures 3A and B show the calculated mean formaldehyde human cancer risks in five different sampling sites and also for different occupations, respectively. The cancer risk ranged from 9.52×10^-5^ to 1.53×10^-3^, and it was greater than the WHO acceptable cancer risk at 10-6 to 10-5 (1 in 1,000,000). For most of the sampling sites and occupations, the cancer risks were about 100–1000 times higher than the acceptable cancer risk.

**Table 1:** Workplace Environment Information

Hospital pathology laboratory	Type of indoor building materials	Ventilation		Ventilation system efficiency (CFM)	Laboratory volume (m^3^)	Exposure time (h)
		General	Local			
No. 1	Stone and Brick			570	48	6.5
No. 2	Stone and Brick			459	24	5.0
No. 3	Stone and Brick			530	24	6.5
No. 4	Stone and Brick			760	48	5.5
No. 5	Stone and Brick			330	96	8.0

**Table 2:** Comparisons of Formaldehyde Human Cancer Risk with Different Occupations and Sampling Sites

Occupations	Pathologist	Lab technician	Office Worker	Housekeeping	Lab Worker
Hospital Pathology Laboratory					
No. 1	1.2247×10^-5^	7.99131×10^-5^	7.99131×10^-5^	3.07492×10^-6^	7.99131×10^-5^
No. 2	3.5369×10^-5^	2.30779×10^-4^	2.30779×10^-4^	8.8799×10^-6^	2.30779×10^-4^
No. 3	1.6412×10^-5^	1.0709×10^-4^	1.0709×10^-4^	4.1206×10^-6^	1.0709×10^-4^
No. 4	4.3222×10^-6^	2.8201×10^-5^	2.8201×10^-5^	1.0851×10^-6^	2.8201×10^-5^
No. 5	7.3395×10^-5^	4.7889×10^-4^	4.7889×10^-4^	1.8427×10^-5^	4.7889×10^-4^

**Table 3:** Comparisons of Formaldehyde Human Non-Cancer Risk with Different Occupations and Sampling Sites

Occupations	Pathologist	Lab technician	Office Worker	Housekeeping	Lab Worker
Hospital Pathology Laboratory					
No. 1	18.560	18.560	18.56	18.56	18.56
No. 2	53.599	53.599	53.599	53.599	53.599
No. 3	24.872	24.872	24.872	24.872	24.872
No. 4	6.550	6.550	6.550	6.550	6.550
No. 5	111.225	111.225	111.225	111.225	111.225

**Figure 4:**
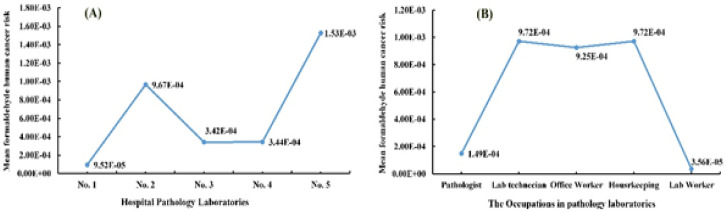
Mean Formaldehyde Human Cancer Risks in Five Different Sampling Sites (A) and Mean formaldehyde human cancer risks in different Occupations (B).

## Discussion

Formaldehyde concentrations in indoor air during tasks ranged from 0.0192 to 0.326 ppm. The indoor formaldehyde levels depended on the concentration in the sources, such as the number of samples, the activities in each sampling site, the temperature and the room’s ventilation. The volume of the pathology departments was 24-96 m^3^, and the recirculation airflow rate was 330-760 CFM.

According to Figure 2, the formaldehyde concentration was higher than the recommended NIOSH exposure limit (REL) for 8 hours (0.016 ppm). However, the results were compared with the OSHA recommended exposure limit (0.75 ppm), and they were lower. The results also showed that the employee exposure was lower than the Iran’s Occupational Exposure Limits (0.3 ppm) in the different sampling sites and occupations, with the exception of hospital No. 2. Similar studies have been carried out in other pathology departments and show high formaldehyde concentrations (higher than NIOSH REL) (Ohmichi et al., 2006) and an increased risk of injuries (Vimercati et al., 2010). Ochs et al., (2011) showed that the personal exposure during the dissection procedure and the mean formaldehyde concentration were higher than the recommended levels. However, Ghasemkhani et al., (2005) depicted that, the formaldehyde concentration in the laboratory pathologies was lower than other places in hospital and was lower than the ACGIH recommended limits because of good local ventilation. 

According to Figures 2A and B, the highest weekly exposure for the five occupations was observed in the department technician (0.646 ppm) and hospital No.2, which could be due to more daily working hours and a lack of adequate ventilation. Also, the lowest weekly exposure was observed in the office workers (0.025 ppm) and hospital No.5, which could be due to the room cavity ratio and the type of tasks involved with office work. According to the Viegas et al., (2010) the occupational exposure of formaldehyde was compared in the anatomy and pathology departments and the formaldehyde-resin production, and the results show that the department employee’s exposure was higher than the formaldehyde-resin production workers. In another study, the formaldehyde concentration was measured in various occupations in Australian workplaces and showed that the pathology department employee’s exposure was medium (Driscoll et al., 2016).

In this study, the formaldehyde human cancer risk for the different occupations and sampling sites was from 1.0851×10^-6^ to 4.7889×10^-4^. According to the results, hospital No.5 had the highest and hospital No.4 had the lowest human cancer risk compared to than others. With regard to occupations, the laboratory workers had the highest and housekeeping had the lowest human cancer risk. With regards to the Cavalcante et al., (2005), there was a greater formaldehyde cancer risk in the female technicians and teaching researchers compared to than other occupations. 

The human non-cancer risk was from 6.55 to 111.225. The lowest human non-cancer risk was related to hospital No.4, and the highest human non-cancer risk also belonged to hospital No.5. With regards to the Costa and co-workers, there was potential health risk in the pathology department employees that were exposed to formaldehyde (Costa et al., 2015). 

The risk classified based on the LCR values in the three levels include a definite risk (LCR less than 10^-4^), probable risks (between 10^-4^ to 10^-5^) and a possible risk (LCR between 10^-5^ and 10^-6^). As a result, there is a risk of cancer (between 10^-4^ and 10^-5^) in hospital No.5, and all of the occupations showed a probable risk. In hospitals No.2 and No.3, the potential risk of cancer in the pathologist and laboratory worker (10^-5^, 10^-6^) was lower than the other occupations. The reason for this is because of less exposure to formaldehyde.

Considering the information collected at the beginning of the research in the pathology department, the department area and the ventilation system efficiency should be appropriate. Based on the effect of these factors in hospital No.5, it is clear that the potential cancer risk is high.

According to the health risk assessment methods in this study, the results of the measurements and assessments can be presented in the health risk levels for the management of chemical exposure and are attributed to the allocation of resources for control actions to reduce the level of exposure risk to formaldehyde in pathology departments.

In Conclusion exposure to formaldehyde has adverse effects on health including both acute and carcinogenic effects. Formaldehyde is widely using in pathology or histology departments of hospitals. This study focused on cancer risk of formaldehyde in pathology department of five hospitals in Rasht, Iran. According to the health risk assessment methods in this study, the results of the measurements and assessments can be presented in the health risk levels for the management of chemical exposure and are attributed to the allocation of resources for control actions to reduce the level of exposure risk to formaldehyde in pathology laboratories. The results of risk assessments can be used for managing chemical exposures of allocated resources for defining control actions. This process playing an important role for reducing the level of exposure to formaldehyde in the pathology departments.
